# Portable Multispectral Optoelectronic System for Thyroid Cancer Detection

**DOI:** 10.3390/s26144448

**Published:** 2026-07-13

**Authors:** Edmilson Roberto Braga, Roberto Márcio Braga Júnior, Mauro Sérgio Braga, Janete Maria Cerutti, Walter Jaimes Salcedo

**Affiliations:** 1 Polytechnic School, Department of Electronic Systems, University of São Paulo (USP), São Paulo 05508-220, SP, Brazil; 2 Center for Exact Sciences, Architecture, and Engineering (CCEAE), Catholic University of Santos (UniSantos), Santos 11015-002, SP, Brazil; robertobraga@unisantos.br; 3 Department of Control and Automation Engineering, Federal Institute of Education, Science and Technology of São Paulo (IFSP), São Paulo 01109-010, SP, Brazil; mauro@ifsp.edu.br; 4 Genetic Bases of Thyroid Tumor Laboratory, Division of Genetics, Department of Morphology and Genetics, Federal University of São Paulo (UNIFESP), São Paulo 04023-062, SP, Brazil; j.cerutti@unifesp.br

**Keywords:** multispectral imaging, transreflectance spectroscopy, immunohistochemistry (IHC), thyroid cancer, biomarkers, supervised machine learning, computer-aided pathology, histopathology

## Abstract

This study reports the development of a portable multispectral optoelectronic system for automated thyroid cancer detection in immunohistochemically stained histological slides. The platform integrates a 14-band AS7343 multispectral sensor, a dual-fiber optical setup operating in transreflectance geometry, and a two-dimensional scanning subsystem for spatially resolved acquisition. Data acquisition and management were implemented on a Raspberry Pi using Python, Flask, React 19.1.0, Redis, and SocketIO for control, visualization, and real-time updates. A standardized Dark–White–Sample protocol was adopted for baseline correction and the generation of multispectral cubes organized by spatial position and spectral band. The dataset comprised 29 patients, 84 FFPE biomarker-stained histological sections, and 66,510 original point-by-point multispectral measurements. Spectral patterns associated with malignant and non-malignant thyroid samples were analyzed using Linear Discriminant Analysis (LDA), Support Vector Machine with radial basis function kernel (SVM-RBF), and Multilayer Perceptron (MLP). All metrics were evaluated on an independent slide-level test set. LDA achieved 86.8% sensitivity, 95.9% specificity, and 91.0% accuracy. SVM-RBF and MLP achieved accuracies of 90.8% and 90.4%, respectively. Macro-averaged AUC-ROC values were 0.836, 0.865, and 0.761, respectively. These findings support the system as a portable proof-of-concept platform for computer-aided thyroid pathology.

## 1. Introduction

Thyroid carcinoma is one of the most prevalent endocrine neoplasms worldwide, with incidence increasing markedly over recent decades. This increase can be partially attributed to advances in imaging modalities that facilitate earlier and more accurate identification of the disease. Thyroid carcinoma is characterized by significant biological heterogeneity, encompassing several histological subtypes, including papillary, follicular, oncocytic, poorly differentiated, undifferentiated or anaplastic and medullary carcinomas [1,2,3,4]. Each of these subtypes presents unique clinical characteristics and prognostic implications, thus complicating the understanding and management of the disease. The process by which normal cells transform into neoplastic cells is a multifactorial phenomenon driven by the accumulation of genetic mutations and epigenetic modifications. These alterations have direct repercussions on essential cellular functions, including cell proliferation, tissue invasion, and tumor aggressiveness [5,6,7].

**Figure 1:**
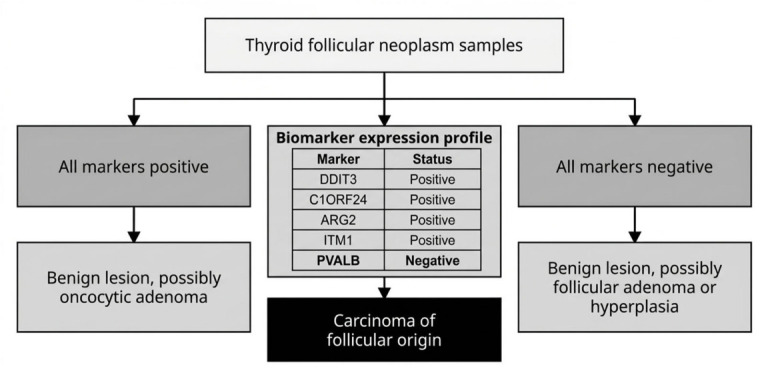
Proposed literature-based decision flow for thyroid follicular neoplasm samples according to the expression patterns of DDIT3, ARG2, ITM1/STT3A, C1orf24/FAM129A, and PVALB [8,9,10,11].

Figure 1 depicts a literature-based decision flow for interpreting the biomarker panel composed of DDIT3, ARG2, ITM1/STT3A, C1orf24/FAM129A, and PVALB for the stratification of thyroid follicular neoplasm samples [8,9,10,11]. This framework supports the biological rationale adopted in the present study for spectral labeling and subsequent computational classification.

Early diagnosis is imperative, as it allows for the implementation of more effective therapeutic strategies, thus improving survival outcomes. In clinical settings, the preoperative diagnosis of thyroid nodules continues to rely predominantly on established techniques such as ultrasound and fine-needle aspiration (FNA) [12,13,14,15]. These methodologies can be further strengthened by incorporating antibody panel-based molecular markers together with the visual evaluation of immunohistochemical (IHC) slides [9,16,17]. Despite the widespread use of IHC, interpretation of the findings remains susceptible to subjective variation, which can impair the reproducibility of diagnostic conclusions [15,17]. In addition to the challenges related to the manual interpretation of IHC samples, particularly in cases of indeterminate cytology, there is a growing demand for more objective, convenient, and economical diagnostic methodologies. These approaches are crucial for improving discrimination between benign and malignant thyroid lesions. The limitations of current diagnostic techniques further complicate the differentiation between benign and malignant neoplasms, increasing patient anxiety, the risks associated with necessary surgical interventions, and the overall financial burden of treatment [16,18].

Despite the use of different biomarkers, as described previously, the IHC diagnostic process, being a microscopy imaging technique limited to three color channels (RGB), may provide indeterminate results; for example, it can fail to resolve borderline follicular neoplasms. Interpretation of staining remains partly subjective, as different pathologists may score the same slide differently, reducing reproducibility. On the other hand, thyroid tumors often show heterogeneous biomarker expression that can lead to inconsistent diagnoses. Conventional IHC is often semi-quantitative, such that it does not provide precise molecular quantification and can vary among observer pathologists [8,9,10,11,15,16,17,18]. Therefore, the search for innovations aimed at improving diagnostic accuracy and enhancing the patient experience is fundamental in the field of thyroid oncology. In this sense, the multispectral thyroid cancer detection system proposed in this work aims to overcome this limitation by quantitative analyses of multispectral maps of samples combined with machine learning tools.

In this context, optical technologies, including multispectral imaging, have emerged as a viable alternative for signal integration in medical diagnosis, particularly for the identification of oncological conditions [19,20,21,22,23]. Beyond the biomedical domain, multispectral sensors have demonstrated versatility across multiple application areas, reinforcing their potential as compact platforms for analytical and diagnostic tasks. In environmental monitoring, for instance, they have been successfully employed in the detection and classification of metallic ions in water bodies, enabling real-time and in situ analysis of contaminants with high sensitivity and selectivity, as demonstrated by portable optoelectronic systems based on colorimetric and fluorescence responses [24,25].

The integration of this technology with artificial intelligence (AI) has shown promising performance, particularly in contexts where differentiation between tissues with similar characteristics is required [23,26,27]. This integration can improve the identification of cancerous tissues and enhance discrimination of tissue patterns that might otherwise remain imperceptible to human observation [26]. Recent advances in AI have further highlighted its importance in refining robust spectral features for the detection of actionable biomarkers and for clinical decision support. A prominent example is the use of these technologies in thyroid cancer during intraoperative procedures, where rapid and reliable decision support is particularly valuable [27]. Neural networks, together with other machine learning (ML) and deep learning (DL) methods, have addressed the challenges associated with manual interpretation of large volumes of spectral imaging data, enabling large-scale intelligent classification with speed and accuracy [26,27,28,29].

We propose a portable multispectral optoelectronic system for thyroid cancer analysis based on a 14-channel sensor, integrated acquisition electronics, spatial traceability, and artificial-intelligence-based pattern recognition. The main contribution of this study is to demonstrate that a compact, lower-cost transreflectance platform can achieve diagnostic performance relative to more sophisticated spectral imaging approaches reported in the literature [19,21,30,31,32,33,34,35,36]. Recent advances in integrated sensing and portable diagnostic platforms further support the feasibility of translating optical and spectroscopic technologies into clinically deployable point-of-care systems [37].

The proposed system translates optical signatures into diagnostic categories grounded in validated biomolecular knowledge from the literature, thereby suggesting a technological basis that can be adapted to other neoplasms that could benefit from spectral differentiation in immunohistochemical slides [19,20].

## 2. Materials and Methods

The methodology for developing and validating the portable multispectral optoelectronic system for thyroid carcinoma detection was structured to ensure reliable and reproducible analytical results, from sample selection to computational modeling and independent test-set evaluation. The dataset comprised 84 formalin-fixed, paraffin-embedded (FFPE) slides obtained from 29 anonymized patients. Only biomarker classification information was available for analysis, while detailed clinical variables were not accessible, and 66,510 original point-by-point multispectral measurements were acquired under the standardized Dark–White–Sample protocol.

### 2.1. Sample Collection

Thyroid tissue samples were collected based on well-established clinical and pathological criteria, encompassing both benign and malignant nodules. Formalin-fixed, paraffin-embedded (FFPE) thyroid tissue sections were obtained from the pathology archives of the Federal University of São Paulo (UNIFESP) from patients who had undergone thyroidectomy for histologically confirmed thyroid carcinoma or benign lesions. Serial FFPE sections from the selected cases were processed using the biomarker panel to support benign or malignant stratification of the thyroid lesions. The final dataset included 84 biomarker-stained histological sections/slides from 29 patients. The biomarkers used were DDIT3, ITM1 (STT3A), ARG2, C1orf24 (FAM129A), and PVALB. Their expression produced distinct staining patterns in the samples according to the biological status of the tissue [8,9,10,11]. The diagnostic procedure was performed by visualizing the sample’s coloration using a conventional microscope, where positive tumor regions were differentiated by applying the chromogen DAB (brown), while negative or stromal areas were delineated by a blue hue in hematoxylin [17,32].

### 2.2. Multispectral Optoelectronic System

**Figure 2:**
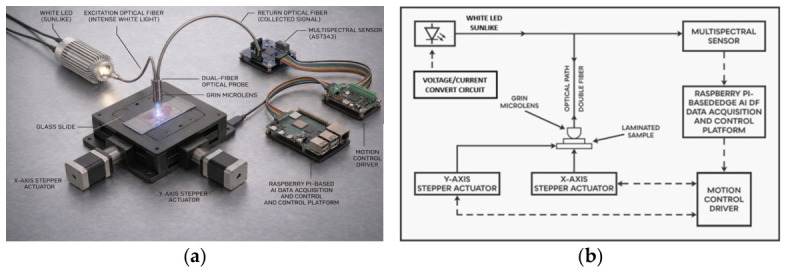
Proposed portable multispectral optoelectronic system for thyroid slide analysis by transreflectance spectroscopy: (**a**) illustrative graphical rendering of the proposed physical setup; (**b**) functional schematic diagram of the acquisition and control architecture.

Figure 2 depicts the proposed portable multispectral optoelectronic system assembled for thyroid slide characterization by transreflectance spectroscopy, including (a) an illustrative graphical rendering of the proposed physical setup and (b) a functional schematic diagram of the acquisition and control architecture. The system architecture incorporates the AS7343 multispectral sensor from ams-OSRAM AG, Premstaetten, Austria, which provides 14 spectral channels spanning the visible and near-infrared range according to the manufacturer’s technical specifications [38], and it was integrated into the proposed acquisition system for optical characterization and multispectral analysis [30,31,32,33,34,35].

**Figure A1:**
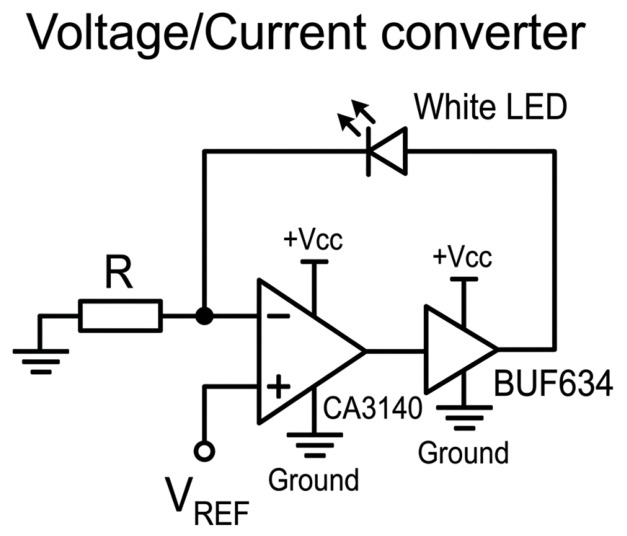
Schematic diagram of the voltage-to-current converter circuit [39].

Data acquisition and preprocessing were performed by the Raspberry Pi 5 system. This hardware board served as the main controller for the acquisition protocol (configuration, synchronization, and recording of measurements). However, to avoid voltage-level incompatibility with the multispectral sensor, the electrical interface was isolated by means of the AdUM4160 device from Mouser Electronics, Inc., Mansfield, TX, USA, with the aim of mitigating conducted interference and parasitic couplings that could impair signal stability. Light excitation was provided by LEDs operating in the visible spectrum, specifically employing a Mid-Power LED—3030 Series from Seoul Semiconductor, Ansan-si, Republic of Korea, characterized as a cool white SMD device with a correlated color temperature (CCT) of 5000 K. This LED provides a broad emission spectrum across the visible range, high luminous efficiency, and good spectral stability, making it suitable for optical sensing applications. Its compact 3.0 × 3.0 mm package and uniform light distribution further support its integration into fiber-coupled systems. The LED source was optically coupled to a fiber that directs the excitation beam onto the sample to be characterized. It is important to note that the luminous intensity of the LED may fluctuate due to temperature variations and the characteristics of the applied driving voltage. To mitigate these effects, a dedicated current control circuit was implemented, as illustrated in Appendix A Figure A1. This circuit was developed using operational amplifiers (CA3140) and buffer stages (BUF634), providing an interface in which the LED intensity exhibits a linear dependence on a reference voltage (VREF), thereby improving repeatability and measurement reliability [39].

The AS7343 multispectral sensor was configured with a high analog gain setting of 2048×, enabling enhanced sensitivity for low-intensity signal detection. The integration time (t_int_) was set to 182 ms, resulting in an optimal trade-off between the signal-to-noise ratio and acquisition time, ensuring stable and reliable spectral measurements under the adopted experimental conditions.

Thus, the excitation beam traverses the tissue section and the substrate, and the retroreflected signal is collected by an optical fiber coupled to the AS7343 multispectral detector. The spatial correlation between the observed morphology and the sampling points is ensured through the use of a USB digital microscope mounted on an XYZ positioning stage, which is driven by dedicated motors and stepper drivers, allowing controlled positioning in designated regions of interest and ensuring traceability between x, y, and z coordinates, pathological point-to-point map, and spectral signatures. It is important to point out that the proposed system is a point-to-point multispectral mapping system that generates a spectral map of the sample with a resolution defined by the size of the excitation light beam (100 µm). Thus, even though it does not characterize a hyperspectral image, the proposed system provides a hyperspectral point-to-point map of the sample. Future developments may incorporate dense spatial sampling and image reconstruction approaches to provide broader spatial coverage of heterogeneous tissue regions.

The data acquisition and management pipeline was implemented in Python 3.13.5, structured as a software service that controls collection, organizes storage, and makes results available to the operator. In architectural terms, Flask was used for the server layer (responsible for receiving commands, triggering acquisition routines, and displaying the data), React for the graphical interface (through which the user monitors and controls the process), and Redis/SocketIO components were used to enable real-time updates between the system and the interface, allowing acquisition progress and measured values to be displayed in real time. The measurement protocol follows a predetermined sequence composed of three steps, designed to standardize acquisition and reduce instrumental biases. In the Dark step (LED off), the system’s background signal (electronic noise and residual sensor contribution) is recorded, which is subsequently used for baseline correction. Then, in the White step (clean reference area), a reading is taken in a stable optical reference region, representative of the system’s response under nominal illumination and the behavior of the substrate/screen in the absence of tissue sample, allowing for the normalization of intensities per channel and compensation for global variations in illumination and coupling. Finally, in the Sample stage, spectral signatures are collected at the area of interest in the tissue on the slide sample. The spectral readings are standardized through channel-by-channel normalization, with the aim of making measurements performed on different slides and acquisition conditions comparable such that the response pattern corresponds to the spectral structure of the transreflectance, thus avoiding fluctuations due to intensity variations. Let D(λ) denote the dark reading acquired with the emitter off, representing the system background level, including electronic noise, at wavelength λ. Let S(λ) denote the raw signal measured in the region of interest of the sample. Let W(λ) denote the white-reference reading used as an internal illumination and return standard. The spectral normalization, expressed as transreflectance, is given by

(1) $$R\left(\lambda \right)=\frac{S\left(\lambda \right)-D\left(\lambda \right)}{W\left(\lambda \right)-D\left(\lambda \right)}$$

From these normalized spectra, the data are organized into multispectral cubes, in which two dimensions represent the spatial scan over the slide (x and y coordinates) and the third dimension represents the spectral bands (channels). This representation simultaneously structures spatial and spectral information, serving as the basis for subsequent feature extraction steps, in which spectral descriptors and indices are derived to feed the analytical and classification models.

### 2.3. Data Acquisition and Processing

Data acquisition and processing were carried out in the following steps.

#### 2.3.1. Acquisition, Integration, and Initial Cleaning of Multispectral Data

In the acquisition phase, experimental procedures were performed based on two main categories of information retained in the multispectral optoelectronic platform database: (i) a compilation of metadata relating to slides and acquisitions (e.g., slide identification, biomarker designation, acquisition status, and temporal specifications) and (ii) a dataset encompassing point-by-point spectral measurements, documented as multichannel intensities of the transreflectance signal captured in back-collection geometry (where illumination and collection occur on the same side of the sample) within the visible spectrum. The metadata and measurements were merged into a unified dataset in order to correlate, for each measured point, information about the biomarker, the corresponding slide, the acquisition context, and the specific location on the slide, thereby preserving valid measurements and enriching each record with attributes critical for subsequent analyses. Finally, intra-slide normalization was employed to mitigate the absolute scaling effects resulting from variations between slides and acquisition conditions: for each slide, the transreflectance signal intensities within a reference category (e.g., regions designated as internal standard) were used to normalize other channel-to-channel measurements, scaling each band relative to a maximum reference value of the slide itself and producing relative intensity measurements, which reduces the impact of global variations in illumination, tissue thickness, and optical coupling, thereby facilitating more equitable comparisons within regions and between different slides [35,36].

#### 2.3.2. Exploratory Analysis and Quality Diagnosis

During the exploratory analysis phase, after normalization, methodical evaluations were conducted on spectral channels. For each spectral band, descriptive statistics—including minimum, maximum, mean, and standard deviation—were calculated, and empirical distributions were evaluated with the aim of detecting extreme values that might indicate saturation or sensor artifacts, observing asymmetries and dispersions that could suggest spectral heterogeneity between tissue regions, and identifying bands that were not informative or were predominantly influenced by noise for possible exclusion. Simultaneously, diagnostic protocols were established with three complementary focal points: (1) the physical plausibility of the data, verifying whether the normalized intensities remained within ranges that align with the platform’s operational parameters and the predicted optical characteristics of biological tissues within the specified spectral range; (2) the integrity of the fundamental truth table, which involved verifying the integrity and consistency of the binary (positive/negative) labels associated with each biomarker on individual slides, ensuring conformity with established histopathological reference knowledge; and (3) an initial examination of the correlation between the model outputs and the labels, analyzing how continuous scores and predicted probabilities correlated with the true labels, in order to proactively identify limitations in spectral separability and highlight biomarkers with inherently more complex patterns. These assessments served as a methodological quality control, ensuring that the data designated for supervised training were not only physically plausible but also semantically consistent with the detection task associated with the slides labeled with PVALB, ARG2, ITM1, DDIT3, and C1ORF24 [37,38,40,41,42].

#### 2.3.3. Spectral Preprocessing and Noise Cleaning

In the preprocessing phase, after intra-slide normalization, a systematic procedure was performed to reduce noise and instrumental artifacts, ensuring that the spectral signatures of the transreflectance signal used in supervised training primarily reflected variations attributable to tissue and biomarker expression (PVALB, ARG2, ITM1, DDIT3, and C1ORF24), with minimal interference from random fluctuations, saturation, and system instabilities [42,43,44,45,46,47].

#### 2.3.4. Dataset Partitioning, Synthetic Data Generation, and Independent Test Evaluation

After preprocessing and quality control, the original dataset comprised 66,510 point-by-point multispectral measurements obtained from 84 biomarker-stained FFPE histological slides/sections, corresponding to 84 regions of interest from 29 patients. To increase the representation of spectral variability during model development, 336,960 synthetic multispectral measurements were generated from the original measurements, resulting in a total analytical dataset of 403,470 multispectral measurements when the original and synthetic data were considered together.

Data partitioning was performed at the slide level, so that all multispectral measurements associated with a given slide remained exclusively within a single subset. This strategy prevented point-level leakage between training and testing and ensured that measurements originating from the same histological slide were not shared across model-development and evaluation stages. Synthetic data generation was conducted after respecting the slide-level partitioning structure and was restricted to the model-development workflow, without using test-set information for model fitting, hyperparameter adjustment, or threshold selection. Accordingly, the independent test set remained separated from training and optimization procedures, preserving an unbiased assessment of out-of-sample classifier performance.

### 2.4. Machine Learning Classifiers for Biomarker Detection in Multispectral Data

The classification step plays a central role in the processing pipeline of the portable multispectral optoelectronic system for thyroid cancer detection, as it is in this step that the spectral signatures measured in each slide region are finally converted into objective decisions of positivity or negativity for the biomarkers PVALB, ARG2, ITM1, DDIT3 and C1ORF24. In the context of the present study, the central task consists of analyzing, for each region of interest in the histological slides, the spectral transreflectance signatures and deciding whether the spectral pattern associated with each biomarker should be classified as positive or negative. Each region of the slide is therefore represented by a vector of transreflectance intensities at different wavelengths in the visible spectrum, captured by the portable optoelectronic system. These vectors translate, in quantitative terms, the optical patterns associated with the local expression of each biomarker in benign or malignant thyroid tissues, constituting the basis on which supervised learning models operate [48,49,50,51]. The implementation of machine learning algorithms, such as Support Vector Machines and neural networks, can improve the classification of spectral signatures, supporting more reliable and efficient diagnostic workflows.

Given a scenario characterized by high dimensionality (multiple strongly correlated wavelengths), interlayer variability, and a limited number of labeled samples, it becomes essential to employ models capable of complementarily exploring both linear and nonlinear structures present in the data. For this purpose, three consolidated supervised classification paradigms are considered: Linear Discriminant Analysis (LDA), which represents a parsimonious linear probabilistic approach; Multilayer Perceptron (MLP), which introduces the ability to model complex nonlinear relationships through multilayer neural networks; and Support Vector Machine (SVM), which relies on margin maximization and kernel functions to capture potentially nonlinear decision boundaries in high-dimensional spaces [52,53,54,55,56]. Section 2.4.1, Section 2.4.2 and Section 2.4.3 detail, respectively, the mathematical formulation, the implicit assumptions, the regularization strategies and the practical implementation aspects of each of these classifiers in the specific context of multispectral slide analysis for thyroid cancer detection. Even though hyperspectral image processing and analysis conventionally utilize AI methods related to convolutional neural networks, in our case, because the data correspond to point-to-point multispectral maps, the data processing and analysis were better suited to LDA, SVM, and MSP AI.

#### 2.4.1. Linear Discriminant Analysis (LDA)

In the context of quantitative analysis of histological images and optical spectroscopy, Linear Discriminant Analysis (LDA) constitutes a classic and widely used approach for binary decision problems, especially in scenarios with high dimensionality and a limited number of labeled samples. In this work, LDA is employed to determine, in each slide region, whether the spectral pattern associated with each biomarker (PVALB, ARG2, ITM1, DDIT3, C1ORF24) should be interpreted as positive or negative, based on spectral transreflectance data acquired at different wavelengths of the visible spectrum for each spatial region of interest [57,58,59,60,61].

From these signatures, LDA produces a linear decision rule capable of separating positive and negative regions for each biomarker by exploiting systematic differences in transreflectance curves [59,62].

In the binary formulation, LDA seeks a discriminant direction in the feature space along which the separation between the distributions of positive and negative signatures is maximized [59,63,64,65]. This direction is represented by the discriminant vector, given, up to proportionality, by

(2) $$w\propto \Sigma^{-1}\left(\mu_{1}-\mu_{0}\right)$$

where μ_1_ and μ_0_ are the mean vectors of the spectral signatures of the positive and negative regions, respectively, and Σ is the covariance matrix shared between the classes. The difference vector encodes the average shift in the spectral signatures when moving from negative to positive regions, while multiplication by Σ^−1^ corrects this direction by taking into account the variability and correlations among the different transreflectance bands.

In interpretative terms, each component of w indicates the relative weight of a given wavelength range in discriminating between positive and negative regions of a biomarker. The corresponding linear discriminant function is defined as

(3) $$g\left(x\right)=w^{T}x+b$$

where x is the standardized spectral feature vector and b is the bias term. Estimation of the covariance matrix plays a central role in the quality of the LDA solution. In visible spectroscopy data, two characteristics stand out: (i) adjacent spectral bands tend to be strongly correlated, producing collinearity between features; and (ii) the number of labeled samples is small relative to the number of bands. Under these conditions, the empirical covariance matrix may be ill-conditioned or nearly singular, making its inversion difficult and rendering the classifier extremely sensitive to noise and sample variations [66,67]. These challenges highlight the importance of implementing robust cross-validation and feature selection techniques to ensure the effectiveness of machine learning models in clinical settings.

To circumvent this problem, a regularized estimate of the covariance is adopted in the form

(4) $$\Sigma_{reg}=\left(1-\alpha \right)\Sigma_{emp}+\alpha I$$

where *Σ_emp_* is the empirical covariance matrix calculated from the transreflectance intensities, I is the identity matrix, and α is an automatically chosen shrinkage parameter.

This convex combination of *Σ_emp_* and I shrinks the covariance towards a simpler, spherically symmetric model, reducing the impact of spurious correlations and improving the numerical conditioning of the matrix. As a consequence, inversion of the covariance matrix becomes more stable, and the discriminant vector more robustly reflects the real patterns of spectral variation relevant to the classification task.

Before model fitting, spectral features are standardized so that each transreflectance band has a mean of zero and a variance of one. This step prevents spectral bands with higher numerical variance from dominating the estimation of the class means and covariance structure simply because they are on a different scale, rather than because they are more informative. By working with standardized data, all bands contribute on a comparable scale; thus, LDA can effectively exploit mean differences and correlation patterns between classes, instead of primarily capturing artificial scale differences [67,68].

Once the discriminant vector and bias term have been estimated, each standardized sample x, which corresponds to the transreflectance vector of a slide region, is projected along the discriminant direction. The resulting discriminant score is given by g(x), as defined in Equation (3).

This score represents the sample’s position along the axis that best separates the typical spectral signatures of positive and negative regions for the biomarker in question. High values indicate greater similarity to the positive spectral pattern estimated by the model, while low values indicate greater similarity to the negative pattern. The decision displayed to the user (positive or negative classification of the region) is obtained by comparing this score to a threshold. Under the Gaussian assumptions of LDA, this same score can be associated with posterior class probabilities, which allows not only categorical decisions but also a measure of confidence [57,68].

Thus, the positivity or negativity decisions for PVALB, ARG2, ITM1, DDIT3 and C1ORF24 biomarkers are based on a well-established linear statistical model that integrates probabilistic assumptions, high-dimensional regularization and adequate preprocessing of transreflectance signatures. This framework offers a favorable compromise between interpretability, numerical robustness and generalization capacity for new slides and acquisition conditions [69,70,71,72]. The implementation of LDA, in conjunction with other machine learning techniques, can improve not only diagnostic accuracy but also facilitate the identification of complex patterns in spectral signatures, thus optimizing the diagnosis of thyroid carcinoma.

#### 2.4.2. Support Vector Machine with RBF Kernel (SVM-RBF)

Among the reference classifiers in the machine learning literature, Support Vector Machines (SVMs) stand out in scenarios characterized by moderate/high dimensionality, a limited number of labeled samples, and possibly nonlinear decision boundaries, typical conditions for studies with biomarkers in histological slides. In this approach, for each biomarker, a binary classifier is trained that receives as input the spectral transreflectance vector of a region of the slide and produces as output a positive or negative label, reflecting the presence or absence of the spectral pattern compatible with the expression of that molecular target [73,74,75].

SVM is formulated as a maximum margin classifier. In its canonical form, SVM searches for the hyperplane that separates the classes in order to maximize the margin, that is, the distance between the hyperplane and the nearest samples of each class (the support vectors). This maximization of the margin is directly linked to better generalization properties in limited datasets, reducing the tendency to overfit, which is particularly relevant in this study, where the number of annotated slide samples and regions is restricted, but the number of spectral attributes is sufficient to make the problem non-trivial. In practical terms, this implies that the model’s decision is governed by a subset of “critical” samples, located on the boundary between spectral patterns of regions considered positive and negative, providing robustness to noise and variations in regions further away from this boundary [76,77,78,79,80].

To capture the nonlinearity expected in the separation between transreflectance profiles associated with different histopathological states, a Gaussian radial basis function (RBF) kernel was adopted. This choice is also consistent with previous applications of SVM-based classifiers to spectral and hyperspectral datasets, including near-infrared microscopy libraries, hyperspectral tissue classification, Raman spectroscopy, and other high-dimensional spectroscopic classification problems, in which margin-based learning has been used to improve discrimination in complex biomedical and analytical spectra [81,82,83,84,85].

During inference, the SVM with RBF kernel produces, for each spectral vector associated with a region of the slide, a scalar value corresponding to the decision function. This value approximately represents the signed distance to the separating hyperplane in the feature space induced by the kernel: f(x) > 0 indicates that the region is on the side associated with the positive class for that biomarker, whereas f(x) < 0 indicates the side associated with the negative class. The magnitude |f(x)| provides a geometric measure of confidence: regions farther from the boundary between the typical spectral distributions of positive and negative samples tend to exhibit more stable decisions.

In the dataset studied, this SVM-based approach proved suitable for capturing spectral patterns associated with biomarker expression in benign and malignant regions, producing competitive performance in terms of the balance between sensitivity and specificity, as detailed in Section 3.

#### 2.4.3. Multilayer Perceptron (MLP)

As a third approach in this study, a Multilayer Perceptron (MLP) neural network was used for binary classification in each slide region of the biomarkers PVALB, ARG2, ITM1, DDIT3, and C1ORF24 as positive or negative. As with the previous classifiers, each region is represented by a vector of light transreflectance intensities at different wavelengths of the visible spectrum, measured by the portable optoelectronic system, so that the network operates directly on the spectral signatures associated with benign and malignant regions [86,87].

From a technical standpoint, dense neural networks of the MLP type constitute a flexible and widely used model in problems with nonlinear relationships between attributes and the target variable, including applications in spectroscopy, biomedical signal analysis, medical hyperspectral and optical imaging, and medium-dimensional tabular data [88,89,90,91,92]. While LDA and SVM with RBF kernel already offer, respectively, a regularized linear model and a maximum margin classifier with nonlinear boundaries, MLP adds the ability to learn highly nonlinear composite mappings through multiple layers of neurons with nonlinear activation functions. In practical terms, this allows the model to capture more complex combinations between transreflectance bands that may be associated with subtle expression patterns of biomarkers in thyroid tissues [87].

The adopted architecture consists of two dense hidden layers with 64 and 32 neurons, respectively, both using the ReLU (Rectified Linear Unit) activation function, followed by an output layer with a single neuron and sigmoid activation. The ReLU functions enable the model to efficiently approximate nonlinear functions, while avoiding some of the saturation and very small gradient problems associated with sigmoid or Tanh (Hyperbolic Tangent) activation functions in inner layers [93,94,95,96,97]. The choice of two hidden layers with 64 and 32 neurons reflects a compromise between modeling capability (sufficient to capture complex spectral patterns) and parsimony, given the limited number of samples labeled by biomarker. The output neuron with sigmoid activation produces a continuous value, interpreted as a positive class probability, which is naturally suitable for the task of binary classification of regions as positive or negative. The use of compact dense architectures operating directly on spectral feature vectors is also supported by recent deep learning studies on spectral data modeling and spectral quantification, which highlight the influence of input representation, network architecture, optimization strategy, and training bias on model performance [98,99].

Network training is performed using the Adam optimizer, which combines ideas of momentum and adaptive learning rate, and is widely accepted in the literature as a robust choice for neural networks on tabular and spectral data [100]. The adaptive learning rate allows the algorithm to automatically adjust the step size in the parameter space, accelerating convergence in stable directions and reducing oscillations in noisier directions [100]. Binary cross-entropy is used as the cost function, which is the standard choice for probabilistic binary classification problems, as it strongly penalizes predictions with high confidence assigned to the incorrect class and is directly related to maximizing the likelihood under the model [101,102].

To reduce the risk of overfitting, especially relevant in neural networks with a larger number of parameters than linear classifiers, two regularization mechanisms are employed. First, an L2 regularization term is used, which adds a penalty proportional to the square of the network weights to the cost function. This term discourages very large weights, favoring smoother solutions and reducing the model’s sensitivity to local variations in the data, which improves the ability to generalize to new slides and regions. Second, the early stopping technique is adopted: a fraction (10%) of the training set is reserved for internal validation, and training is automatically stopped if there is no improvement in the validation metric for 20 consecutive epochs, with a maximum limit of 500 epochs. This procedure prevents the network from continuing to adjust to noise or particularities of the training set after it has already reached its best performance on unseen data, acting in practice as an additional form of regularization [103,104,105].

Once trained, the MLP provides, for each spectral vector corresponding to a region of the slide, a positive class probability obtained directly from the sigmoid output of the final neuron. This probability is used both for binary decision-making (applying a threshold, approximately 0.5, to classify the region as positive or negative) and for constructing risk/certainty maps along the slide, allowing visualization of regions with greater or lesser evidence of expression of the biomarkers PVALB, ARG2, ITM1, DDIT3, and C1ORF24. Unlike SVM, which requires additional calibration to produce well-interpretable probabilities, the MLP is already trained directly to approximate probabilities using binary cross-entropy, which facilitates the clinical/visual interpretation of the model outputs [103,104,105].

In this context, this MLP-based approach complements the LDA and SVM classifiers by offering a model with greater nonlinear flexibility, capable of capturing more complex interactions between transreflectance bands that may be associated with morphological and biochemical variation in the slides. In the experiments performed, the MLP showed competitive performance in terms of accuracy, precision, sensitivity and F1, particularly in biomarkers whose spectral signatures exhibit more complex patterns, as discussed in Section 3. Thus, the inclusion of the MLP in the study not only explores a current trend in the literature to employ neural networks in biomedical problems, but also demonstrates, empirically, that this family of models is able to effectively leverage the information contained in multispectral transreflectance signatures to assist in distinguishing between regions associated with benign and malignant patterns in thyroid cancer [20,29,54].

## 3. Results

This section presents the results obtained with the portable multispectral optoelectronic platform developed in this study for the analysis of thyroid histopathological slides, covering system performance from instrumentation to the generation of pre-classification analytical outputs. The findings reflect the integrated operation of the acquisition module (multispectral sensor, controlled illumination, and automated protocol), the positioning and region-of-interest traceability subsystem, and the computational control and storage layer responsible for recording, organizing, and displaying measurements in real time. At the data level, the results derive from the Dark–White–Sample sequence used for baseline correction and normalization, followed by the organization of measurements into multispectral cubes and the derivation of chromogen-oriented spectral descriptors. From this standardized dataset, the following results report the quantitative performance of the supervised models used to discriminate between positivity and negativity for the biomarkers of interest.

This section summarizes the findings obtained from the evaluation of three classifiers: Linear Discriminant Analysis (LDA), Support Vector Machine with a radial basis function kernel (SVM-RBF), and Multilayer Perceptron (MLP). Each classifier was applied to multichannel signatures derived from the transreflectance signal collected in back-collection geometry to perform binary prediction of positivity or negativity for the biomarkers PVALB, ARG2, ITM1, DDIT3, and C1ORF24 in thyroid tissue. It is important to emphasize that all reported metrics were calculated exclusively on the independent slide-level test set, kept fully separate from model training, synthetic data generation used for model development, and hyperparameter optimization, thereby ensuring an unbiased evaluation of out-of-sample performance.

**Figure 3:**
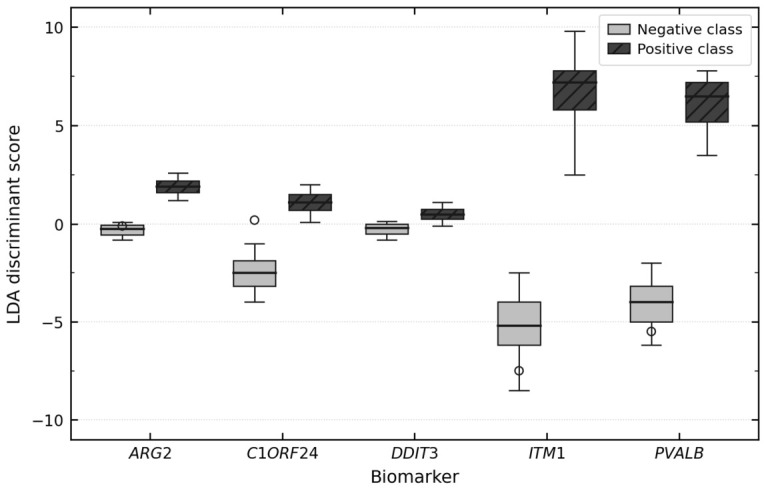
Distribution of LDA discriminant scores across biomarkers and classes.

Figure 3 shows the distribution of LDA discriminant scores by biomarker and class. For all biomarkers, negative-class medians remained below zero and positive-class medians above zero, confirming stable sign-consistent separation under the linear discriminant rule. The largest median gaps were observed for ITM1 and PVALB: ITM1 shifted from a negative-class median of approximately −5.65 to a positive-class median of 6.74, while PVALB shifted from about −3.91 to 6.46. These two biomarkers also showed fully separated interquartile ranges, indicating the most stable linear discrimination among the five targets. ARG2 and C1ORF24 formed an intermediate group, with preserved class ordering but smaller class offsets: ARG2, for example, shifted from a median of about −0.28 in the negative class to 2.07 in the positive class, whereas C1ORF24 shifted from −2.59 to 1.02. DDIT3 showed the smallest effective separation, with a negative-class median near −0.26 and a positive-class median near 0.40, together with the narrowest score dynamic range around zero, indicating the weakest threshold robustness among the five biomarkers under LDA. Although the central separation was strong for most targets, isolated opposite-side outliers remained visible, indicating that a limited subset of samples still departed from the dominant class structure. Overall, the LDA profiles indicate that biomarker performance is governed jointly by median displacement, interquartile overlap, and distance from the zero-threshold region.

**Figure 4:**
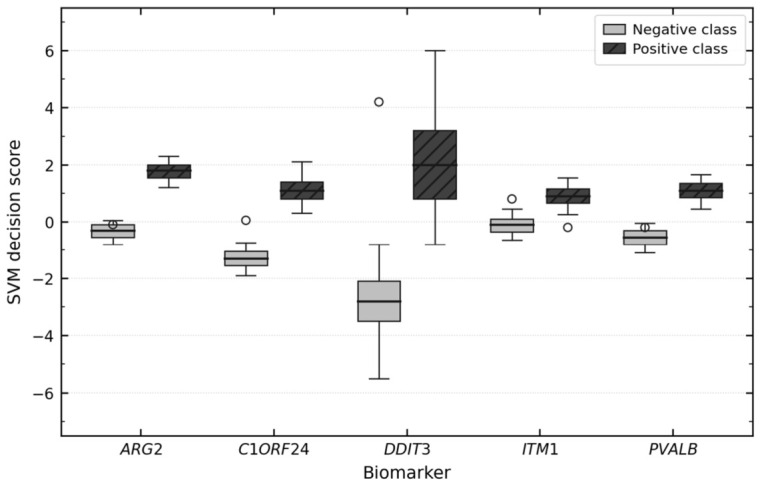
Distribution of SVM-RBF decision scores across biomarkers and classes.

Figure 4 presents the distribution of SVM-RBF decision scores by biomarker and class. For all biomarkers, negative-class medians remained below zero and positive-class medians above zero, confirming stable class orientation under the nonlinear decision function. DDIT3 exhibited the largest median displacement, with the negative class centered near −3.04 and the positive class near 1.95; however, it also showed by far the broadest dispersion, with a negative-class span extending from about −6.07 to 0.90 and a positive-class span from approximately −2.52 to 5.99. This indicates that the strongest central separation was accompanied by the lowest local stability within that model. In contrast, ARG2, C1ORF24, ITM1, and PVALB showed smaller but substantially more compact distributions. ARG2 shifted from a median near −0.18 to 1.79, C1ORF24 from −1.40 to 1.11, ITM1 from −1.15 to 0.99, and PVALB from −0.76 to 1.29, all with lower interquartile spread than DDIT3. These profiles indicate that, under SVM-RBF, margin quality cannot be interpreted from median separation alone: DDIT3 maximized central offset, whereas ARG2, C1ORF24, ITM1, and PVALB achieved more regular discrimination through greater score compactness and reduced tail expansion. The residual opposite-side outliers, especially in DDIT3, reinforce that nonlinear separation improved class contrast but did not fully eliminate biomarker-specific heterogeneity.

**Figure 5:**
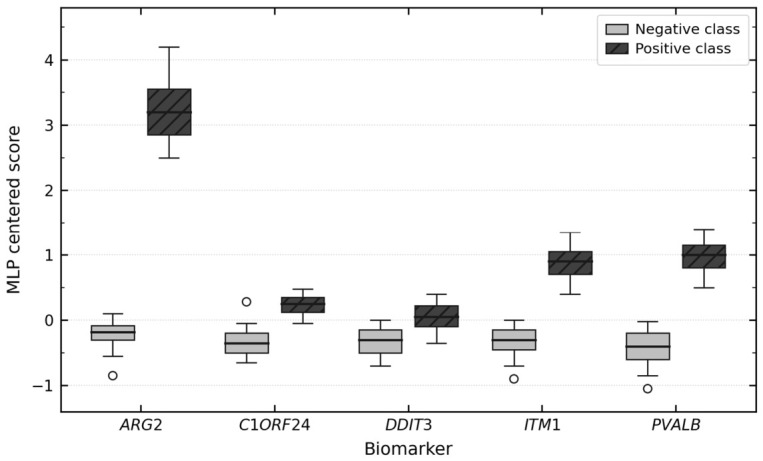
Distribution of MLP-centered scores across biomarkers and classes.

Figure 5 shows the distribution of MLP-centered scores by biomarker and class. Negative-class medians remained below zero and positive-class medians above zero for all five biomarkers, confirming preserved class orientation after nonlinear score centering. ARG2 showed the strongest effective separation, with the negative class centered near −0.29 and the positive class near 3.11, together with broad but clearly disjoint central distributions, making it the most distinctly separated biomarker under the MLP. ITM1 and PVALB formed a second tier, with negative-to-positive median shifts from approximately −0.46 to 0.86 and from −0.64 to 1.00, respectively, and with limited interquartile overlap. C1ORF24 showed a smaller median displacement, from about −0.51 to 0.26, whereas DDIT3 exhibited the narrowest effective margin, with medians close to −0.53 and 0.07 and substantial concentration of both classes around the zero-centered region. Quantitatively, these two biomarkers therefore showed the weakest threshold robustness under the MLP, even though class ordering remained correct. This ranking indicates that MLP discrimination was strongest for ARG2, intermediate for ITM1 and PVALB, and weakest for C1ORF24 and especially DDIT3, which helps explain how the model can remain competitive in global accuracy while still showing biomarker-dependent fragility in score stability and threshold sensitivity.

Within this validation framework, the confusion matrix was used as the main formalism to characterize classifier decision behavior at the selected operating point, partitioning predictions into true positives (TPs), true negatives (TNs), false positives (FPs), and false negatives (FNs). This parameterization defines binary classification performance in this context, directly supporting the calculation of sensitivity, specificity, positive predictive value (PPV), negative predictive value (NPV), and F1-score, while also allowing quantification of error asymmetry between classes. In biomedical research, this analysis is particularly important because false positives and false negatives have different practical consequences. Accordingly, the confusion matrix links statistical metrics to clinical priorities, such as screening versus confirmation, and also enables stratified evaluation by biomarker and/or slide to explore variability in separability and error sources related to tissue heterogeneity and pre-analytical factors.

**Figure 6:**
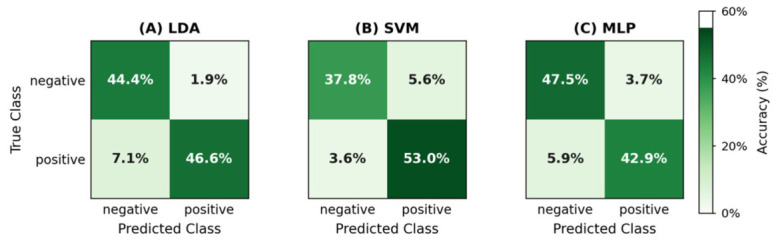
Normalized confusion matrices for the (**A**) LDA, (**B**) SVM-RBF, and (**C**) MLP classifiers on the independent test set.

Figure 6 presents the normalized confusion matrices for the three classifiers on the independent test set, allowing direct visualization of class-specific prediction behavior and error distribution. In all models, most samples were correctly assigned, although distinct error patterns were observed between positive and negative classes, reinforcing the idea that classifier behavior should not be interpreted solely on the basis of global accuracy.

**Figure 7:**
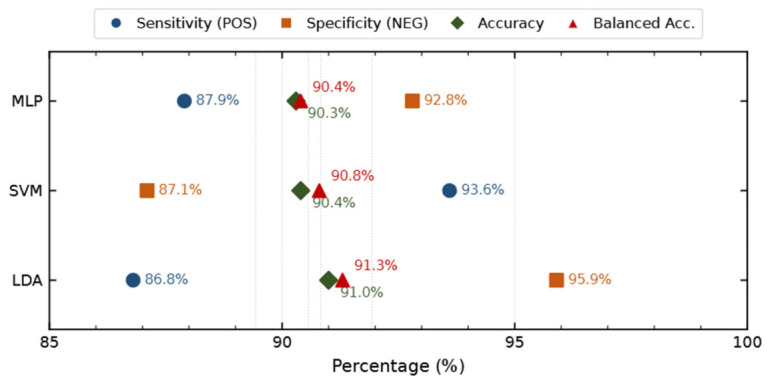
Comparison of sensitivity, specificity, accuracy, and balanced accuracy among LDA, SVM-RBF, and MLP on the independent test set.

Figure 7 compares four key performance metrics across the three classifiers: sensitivity, specificity, overall accuracy, and balanced accuracy. LDA achieved the highest specificity (95.9%) and the highest overall accuracy (91.0%), indicating stronger discrimination of negative cases. SVM-RBF yielded the highest sensitivity (93.6%), favoring positive-case detection at the expense of lower specificity (87.1%). MLP showed a more intermediate profile, with 87.9% sensitivity, 92.8% specificity, 90.4% accuracy, and 90.3% balanced accuracy. Overall, the three classifiers exhibited similar global performance, differing mainly in the sensitivity–specificity trade-off, which may guide model selection according to screening-oriented or confirmatory diagnostic priorities.

Considering all regions of interest and biomarkers jointly, the three models showed competitive overall performance for the supervised analysis of biomedical spectral data. On the independent test set, overall accuracy reached 91.0% for LDA, 90.8% for SVM-RBF, and 90.4% for MLP, while the corresponding F1-scores were approximately 0.906, 0.904, and 0.899. These results reinforce LDA as a strong linear baseline, identify SVM-RBF as the most sensitivity-oriented model among the three, and suggest that the MLP may offer advantages in scenarios involving more complex and potentially nonlinear spectral patterns.

**Figure 8:**
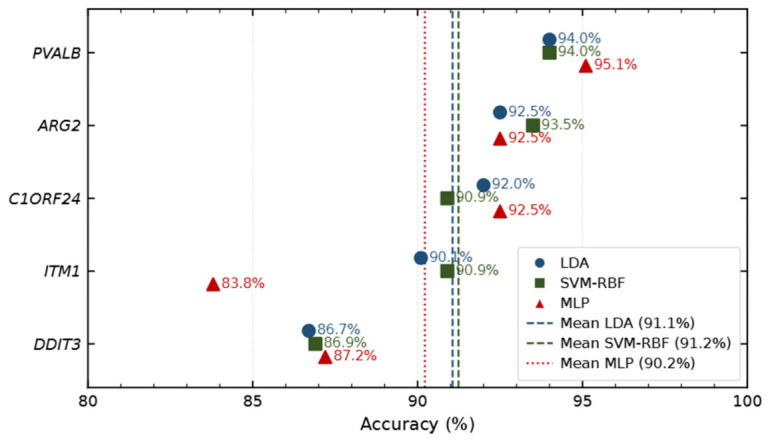
Biomarker-wise classification accuracy across LDA, SVM-RBF, and MLP.

Figure 8 summarizes the final prediction accuracy achieved for each biomarker across the three classifiers. PVALB and ARG2 showed consistently high accuracy across models, whereas DDIT3 yielded the lowest accuracy values overall. The most notable model-dependent difference was observed for ITM1, for which MLP underperformed relative to LDA and SVM-RBF. In combination with the score distributions shown in Figure 4, Figure 5 and Figure 6, these results indicate that final accuracy is shaped not only by the separation between class centers, but also by score dispersion, outlier behavior, and biomarker-specific heterogeneity.

The biomarker-specific comparison further indicates that model choice influences performance unevenly across targets. In particular, the relatively lower ITM1 accuracy obtained with MLP suggests greater sensitivity of this classifier to local variability, whereas PVALB and ARG2 remained consistently well discriminated across all three models. Taken together with the score distributions in Figure 3, Figure 4 and Figure 5, the confusion matrices in Figure 6, and the global metrics in Figure 7, these findings indicate that the purpose of the present comparative analysis is not merely to identify a single universally superior classifier, but to determine which modeling strategy is most appropriate for a given diagnostic priority. In this context, LDA favors more specific and globally accurate decisions, SVM-RBF provides the strongest threshold-independent discrimination and the most sensitivity-oriented profile, and MLP serves as a flexible nonlinear alternative with biomarker-dependent advantages and limitations. Accordingly, classifier selection for future computer-aided thyroid pathology should be guided by intended clinical use, tolerated error profile, and biomarker-specific robustness rather than by overall accuracy alone. The observed differences among biomarkers may be attributed to variations in spectral separability, staining heterogeneity, and biomarker-specific dataset composition. Biomarkers exhibiting more distinctive spectral signatures tended to achieve higher classification performance.

Considering that the receiver operating characteristic (ROC) curve and the area under the ROC curve (AUC) are widely recognized tools for evaluating binary classification performance, we present below the performance of the AI models employed in this study in terms of their respective ROC curves and AUC values.

**Figure 9:**
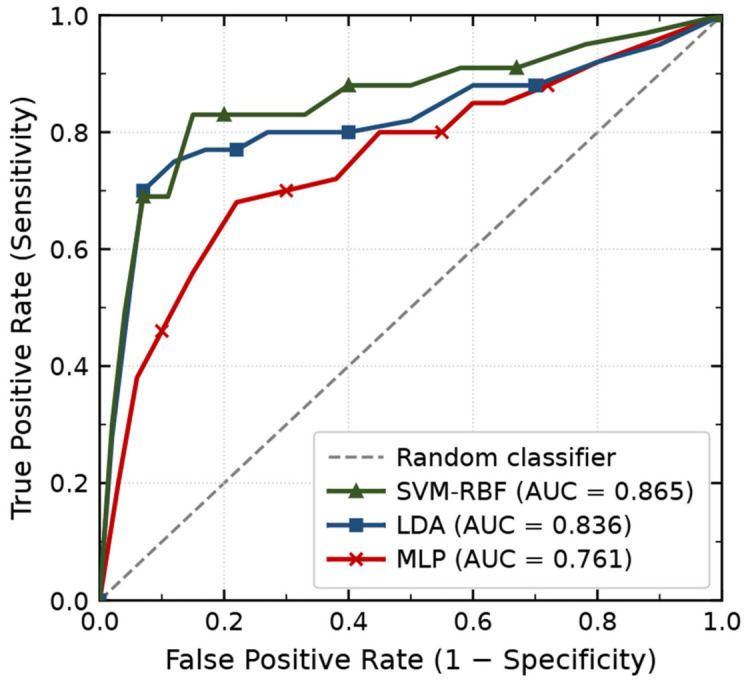
Macro-averaged ROC curves comparing the threshold-independent discriminative performance of LDA, SVM-RBF, and MLP on the independent test set.

Figure 9 shows the macro-averaged ROC curves obtained for the three classifiers on the independent test set. Although LDA, SVM-RBF, and MLP achieved similar overall accuracies, the AUC-ROC analysis revealed clearer differences in threshold-independent discrimination. SVM-RBF achieved the highest AUC-ROC (0.865), outperforming LDA (0.836) and MLP (0.761), indicating a stronger ability to rank positive regions above negative regions across operating thresholds. LDA also maintained competitive discrimination, supporting the presence of linearly exploitable spectral information in the multispectral bands. In contrast, the lower AUC-ROC of MLP suggests reduced robustness in probability ranking under the present experimental conditions. These findings reinforce that classifier selection should consider not only accuracy but also threshold behavior, interpretability, and intended diagnostic use. Although MLP achieved classification accuracy comparable to LDA and SVM, its lower ROC-AUC suggests a reduced ranking capability across the full probability spectrum. This behavior may be associated with probability calibration rather than reduced classification effectiveness at the selected decision threshold.

Appendix A Table A1 presents the training, validation, and test performances of the LDA, SVM-RBF, and MLP classifiers. The close agreement in performance across the three datasets demonstrates satisfactory generalization and provides evidence that substantial overfitting did not occur during model development.

## 4. Discussion

Taken together, the biomarker-level analyses indicate that multispectral transreflectance combined with supervised classifiers of different complexity levels (LDA, SVM-RBF, and MLP) can effectively discriminate positive and negative regions for PVALB, ARG2, ITM1, DDIT3, and C1ORF24 in thyroid cancer classification. Although performance varied across biomarkers and models, convergent evidence from score distributions, confusion matrices, global metrics, and AUC-ROC supports the robustness of the proposed approach and justifies further validation in larger, more heterogeneous cohorts.

Nevertheless, although synthetic multispectral measurements were used to expand spectral variability during model development, the numbers of original patients, slides, and independently validated biological cases remained limited. The limited size of the original labeled cohort, the inter-slide and intra-slide heterogeneity, and the absence of an external validation cohort constrain the statistical generalizability of the reported performance, particularly under biomarker-dependent spectral variability. Accordingly, the current results should be interpreted as a strong proof of concept rather than as definitive evidence supporting immediate clinical deployment.

Table 1 presents a comparative summary of representative studies and their reported performance metrics. The comparison demonstrates that the proposed portable multispectral system achieves performance levels comparable to those reported by more complex spectral imaging platforms. For example, Ref. [106] reported an accuracy of 88.1%, sensitivity of 87.8%, specificity of 88.4%, and AUC of 0.953 using surface-enhanced Raman spectroscopy (SERS) combined with a convolutional neural network for thyroid cancer diagnosis from fine-needle aspiration samples. Reference [104] achieved an AUC of 0.966 and an F1-score of 0.867 using hyperspectral imaging and deep learning on thyroid histological slides. More recently, Refs. [107,108] reported an accuracy of 90.9%, sensitivity of 91.5%, F1-score of 0.898, and AUC of 0.970 using microscopic hyperspectral imaging and a TimeSformer architecture. In comparison, the proposed portable multispectral platform achieved accuracies of 91.0%, 90.8%, and 90.4% for the LDA, SVM-RBF, and MLP classifiers, respectively. The corresponding sensitivities ranged from 86.8% to 93.6%, while specificities ranged from 87.1% to 95.9%. Although some hyperspectral and Raman-based systems reported slightly higher AUC values, these studies generally relied on substantially more complex instrumentation, including hyperspectral cameras with dozens or hundreds of spectral bands, advanced optical setups, and high computational requirements. In contrast, the system proposed in this work employs a compact 14-channel multispectral sensor integrated into a portable low-cost platform while maintaining competitive diagnostic performance.

## 5. Conclusions

This study presented a portable multispectral optoelectronic system for the analysis of thyroid histopathology slides, operating under a transreflectance (back-collection) geometry with spatial traceability through scanning. The architecture integrates the AS7343 multispectral sensor (14 channels, 380–1000 nm), acquisition control on a Raspberry Pi 5, and an XYZ stage coupled to a USB microscope, enabling the connection of morphology, sampling coordinates, and region-of-interest-level spectral signatures.

The measurement protocol was standardized using a Dark–White–Sample sequence, enabling baseline correction and channel-wise normalization. Outputs were organized as multispectral data cubes, and descriptors were aligned with common chromogens (DAB/hematoxylin).

In supervised testing, LDA, SVM-RBF, and MLP achieved overall accuracies of approximately 91.0%, 90.8%, and 90.4%, respectively, with corresponding F1-scores of approximately 0.906, 0.904, and 0.899. In terms of aggregate discriminative performance, AUC-ROC values of approximately 0.836 for LDA, 0.865 for SVM-RBF, and 0.761 for MLP indicated a general advantage of SVM-RBF when performance was assessed across decision thresholds.

Despite relying on a limited number of spectral channels, the proposed AI-assisted multispectral system achieved results comparable to those reported in studies on spectral imaging and artificial intelligence for thyroid cancer detection and histopathological tissue discrimination [20,21,22,23,29], supporting its potential as a portable proof-of-concept platform for computer-aided thyroid pathology. This positioning aligns with the broader transition of thyroid pathology and computational pathology toward image analysis, explainable artificial intelligence, synthetic histology, stain unmixing, and supervised machine-learning-based tissue classification across multimodal histopathological and spectroscopic datasets [107,109,110,111,112,113]. Overall, the findings indicate that compact multispectral transreflectance systems, when combined with appropriate supervised learning strategies, can provide clinically relevant discrimination of biomarker-associated patterns in thyroid tissue. Future studies should expand the dataset, include external validation cohorts, refine calibration and operating-threshold selection, and investigate the integration of spectral and morphological descriptors in order to strengthen translational and clinical potential.

## Figures and Tables

**Table 1:** Comparison of performance metrics between actual work and the reported literature.

Study	Patients	Samples	Optical Techn.	AI Model	Acc. (%)	Sens. (%)	Spec. (%)	F1	AUC
This work (LDA)	29	84 FFPE-IHC slides	Portable MSI (14 channels)	LDA	91.0	86.8	95.9	0.906	0.836
This work (SVM-RBF)	29	84 FFPE-IHC slides	Portable MSI (14 channels)	SVM-RBF	90.8	93.6	87.1	0.904	0.865
This work (MLP)	29	84 FFPE-IHC slides	Portable MSI (14 channels)	MLP	90.4	87.9	92.8	0.899	0.761
[106]	18	36 FNA samples	SERS	CNN	88.1	87.8	88.4	NR	0.953
[104]	23	33 histological slides	HSI	VGG-19 CNN	NR	NR	NR	0.867	0.966
[107]	NR	65 H&E slides	Microscopic HSI	TimeSformer	90.9	91.5	NR	0.898	0.970
[21]	76	200 thyroid specimens	HSI	CNN	79.0	80.0	79.0	NR	0.900
[108]	44	72 specimens	HSI + Radiomics	Machine Learning	83.0	NR	NR	NR	0.850

Acc.: accuracy, Sens.: sensitivity, Spec.: specificity, AUC: area under curve, NR: Not reported.

## Data Availability

The data supporting the findings of this study are available from the corresponding author upon reasonable request due to institutional, privacy, and ethical restrictions.

## Abbreviations

The following abbreviations are used in this manuscript.

AI	Artificial intelligence
AUC	Area under the curve
DAB	3,3′-diaminobenzidine
FFPE	Formalin-fixed, paraffin-embedded
FNA	Fine-needle aspiration
GUI	Graphical user interface
IHC	Immunohistochemistry
LDA	Linear discriminant analysis
ML	Machine learning
MLP	Multilayer perceptron
NPV	Negative predictive value
PPV	Positive predictive value
ROC	Receiver operating characteristic
ROI	Region of interest
SVM-RBF	Support vector machine with radial basis function kernel

## Appendix A

**Table A1:** Metric values for training, test, and validation corresponding to each model.

Model	Set	Accuracy	Sensitivity	Specificity	F1-Score	AUC
LDA	training	92.4%	88.5%	86.8%	0.918	0.858
	test	91.5%	87.3%	96.1%	0.910	0.842
	validation	91.0%	86.8%	95.9%	0.906	0.836
SVM-RBF	training	94.6%	95.9%	90.2%	0.930	0.905
	test	91.6%	94.1%	87.8%	0.911	0.873
	validation	90.8%	93.6%	87.1%	0.904	0.865
MLP	training	93.5%	90.7%	94.6%	0.927	0.812
	test	91.0%	88.5%	93.2%	0.905	0.772
	validation	90.4%	87.9%	92.8%	0.899	0.761

## References

[1] Baloch Z.W., Asa S.L., Barletta J.A., Ghossein R.A., Juhlin C.C., Jung C.K., LiVolsi V.A., Papotti M.G., Sobrinho-Simões M., Tallini G. (2022). Overview of the 2022 WHO Classification of Thyroid Neoplasms. Endocr. Pathol..

[2] Jung C.K., Bychkov A., Kakudo K. (2022). Update from the 2022 World Health Organization Classification of Thyroid Tumors: A Standardized Diagnostic Approach. Endocrinol. Metab..

[3] Juhlin C., Mete O., Baloch Z.W. (2023). The 2022 WHO classification of thyroid tumors: Novel concepts in nomenclature and grading. Endocr.-Relat. Cancer.

[4] Li Y.-D., Ye Q.-Y., Hu X.-R. (2025). Thyroid Cancer: Pathogenesis, Clinicopathology, Diagnosis, and Management. MedComm.

[5] Prete A., de Souza P.B., Censi S., Muzza M., Nucci N., Sponziello M. (2020). Update on Fundamental Mechanisms of Thyroid Cancer. Front. Endocrinol..

[6] Zarkesh M., Zadeh-Vakili A., Azizi F., Foroughi F., Akhavan M.M., Hedayati M. (2018). Altered Epigenetic Mechanisms in Thyroid Cancer Subtypes. Mol. Diagn. Ther..

[7] Luzón-Toro B., Fernández R.M., Villalba-Benito L., Torroglosa A., Antiñolo G., Borrego S. (2019). Influencers on Thyroid Cancer Onset: Molecular Genetic Basis. Genes.

[8] Cerutti J.M., Delcelo R., Amadei M.J., Nakabashi C., Maciel R.M.B., Peterson B., Shoemaker J., Riggins G.J. (2024). A preoperative diagnostic test that distinguishes benign from malignant thyroid carcinoma based on gene expression. J. Clin. Investig..

[9] Cerutti J.M., Latini F.R.M., Nakabashi C., Delcelo R., Andrade V.P., Amadei M.J., Maciel R.M.B., Hojaij F.C., Hollis D., Shoemaker J. (2006). Diagnosis of suspicious thyroid nodules using four protein biomarkers. Clin. Cancer Res..

[10] Cerutti J.M., Oler G., Delcelo R., Gerardt R., Michaluart P., de Souza S.J., Galante P.A.F., Huang P., Riggins G.J. (2011). PVALB, a new Hürthle adenoma diagnostic marker identified through gene expression. J. Clin. Endocrinol. Metab..

[11] Cerutti J.M. (2011). Employing genetic markers to improve diagnosis of thyroid tumor fine needle biopsy. Curr. Genom..

[12] Haugen B.R., Alexander E.K., Bible K.C., Doherty G.M., Mandel S.J., Nikiforov Y.E., Pacini F., Randolph G.W., Sawka A.M., Schlumberger M. (2016). 2015 American Thyroid Association Management Guidelines for Adult Patients with Thyroid Nodules and Differentiated Thyroid Cancer: The American Thyroid Association Guidelines Task Force on Thyroid Nodules and Differentiated Thyroid Cancer. Thyroid.

[13] Grani G., Sponziello M., Pecce V., Ramundo V., Durante C. (2020). Contemporary Thyroid Nodule Evaluation and Management. J. Clin. Endocrinol. Metab..

[14] Kristollari K., Paul A.A., Angel S., Marks R.S. (2024). Conventional and Emerging Diagnostic Approaches for Differentiated Thyroid Carcinoma. Chemosensors.

[15] Harahap A.S., An S.K., Kim M., Na H., Jang E., Kim H., Jung C.-K., Bychkov A. (2025). Diagnostic challenges in the assessment of thyroid neoplasms using nuclear features and vascular and capsular invasion: A multi-center interobserver agreement study. J. Pathol. Transl. Med..

[16] de Matos L.L., Del Giglio A.B., Matsubayashi C.O., de Lima Farah M., Del Giglio A., da Silva Pinhal M.A. (2012). Expression of CK-19, galectin-3 and HBME-1 in the differentiation of thyroid lesions: Systematic review and diagnostic meta-analysis. Diagn. Pathol..

[17] Crescenzi A., Baloch Z.W. (2023). Immunohistochemistry in the pathologic diagnosis and management of thyroid neoplasms. Front. Endocrinol..

[18] El-Shorbagy S.H., El-Azeem M.A.A. (2015). Differentiation of benign from malignant follicular-cell-derived thyroid lesions: An immunohistochemical study. Egypt. J. Pathol..

[19] Cece A., Agresti M., De Falco N., Sperlongano P., Moccia G., Luongo P., Miele F., Allaria A., Torelli F., Bassi P. (2025). Role of Artificial Intelligence in Thyroid Cancer Diagnosis. J. Clin. Med..

[20] Almagor M., Shapira Y., Soker A., Fischer G., Gartner J., Mai S., Garini Y. (2026). Thyroid cancer detection and classification using spectral imaging and artificial intelligence. Sci. Rep..

[21] Halicek M., Dormer J.D., Little J.V., Chen A.Y., Fei B. (2020). Tumor detection of the thyroid and salivary glands using hyperspectral imaging and deep learning. Biomed. Opt. Express.

[22] Baffa M.F.O., Bachmann L., Pereira T.M., Felipe J.C. Hyperspectral Signal Analysis for Thyroid Neoplasm Typification on Infrared Spectrum. Proceedings of the IEEE 34th International Symposium on Computer-Based Medical Systems (CBMS).

[23] Leitch K., Halicek M., Shahedi M., Little J.V., Chen A.Y., Fei B. (2022). Detecting aggressive papillary thyroid carcinoma using hyperspectral imaging and radiomic features. Proc. SPIE.

[24] Braga M.S., Gomes O.F., Jaimes R.F.V.V., Braga E.R., Borysow W., Salcedo W.J. Multispectral colorimetric portable system for detecting metal ions in liquid media. Proceedings of the 2019 4th International Symposium on Instrumentation Systems, Circuits and Transducers (INSCIT).

[25] Lopin P., Nawsang P., Laywisadkul S., Lopin K.V. (2025). Evaluation of low-cost multi-spectral sensors for measuring chlorophyll levels across diverse leaf types. Sensors.

[26] Nagendra L., Pappachan J.M., Fernandez C.J. (2023). Artificial intelligence in the diagnosis of thyroid cancer: Recent advances and future directions. Artif. Intell. Cancer.

[27] Habchi Y., Himeur Y., Kheddar H., Boukabou A., Atalla S., Chouchane A., Ouamane A., Mansoor W. (2023). AI in Thyroid Cancer Diagnosis: Techniques, Trends, and Future Directions. Systems.

[28] Habchi Y., Kheddar H., Himeur Y., Ghanem M.C. (2026). Machine learning and transformers for thyroid carcinoma diagnosis. J. Vis. Commun. Image Represent..

[29] Baffa M.F.O., Zezell D.M., Bachmann L., Pereira T.M., Deserno T.M., Felipe J.C. (2024). Deep neural networks can differentiate thyroid pathologies on infrared hyperspectral images. Comput. Methods Programs Biomed..

[30] Zufry H., Munawar A.A. (2024). Near-Infrared Spectroscopy for Distinguishing Malignancy in Thyroid Nodules. Appl. Spectrosc..

[31] Tholkappian R., Sinha S., Chinni B., Rao N., Dogra V.S. Computer-aided tissue characterization for detection of thyroid cancer using multi-wavelength photoacoustic imaging. Proceedings of the 2nd International Conference on Paradigm Shifts in Communication, Embedded Systems, Machine Learning and Signal Processing (PCEMS).

[32] Price P., Ganugapati U., Gatalica Z., Alayed M., Abdujabbarov R.S., Sundback C.A., Farris A.M., Turakulov O. (2023). Reinventing Nuclear Histo-score Utilizing Inherent Morphologic Cutoffs: Blue-brown Color H-score (BBC-HS). Appl. Immunohistochem. Mol. Morphol..

[33] Durán-Díaz I., Sarmiento A., Fondón I., Bodineau C., Tomé M., Durán R.V. (2024). A Robust Method for the Unsupervised Scoring of Immunohistochemical Staining. Entropy.

[34] Liao J., Wang Z., Zhang Z., Zhang Z., Bian Z., Guo K., Jiang S., Chen Q. (2018). Dual light-emitting diode-based multichannel microscopy for whole-slide multiplane, multispectral and phase imaging. J. Biophotonics.

[35] La Salvia M., Torti E., Lago G., Leon R., Gandolfi R., Silveri G., Rossella M., Danese G., Lago P., Leporati F. (2023). Hyperspectral imaging acquisition set-up for medical applications. Hyperspectral Imaging and Applications II.

[36] Rosa-Olmeda G., Villa M., Hiller-Vallina S., Chavarrias M., Pescador F., Gargini R. (2024). A Microscope Setup and Methodology for Capturing Hyperspectral and RGB Histopathological Imaging Databases. Sensors.

[37] Zhou M., Li J., Yuan S., Yang X., Lu J., Jiang B. (2024). A centrifugal microfluidic system for automated detection of multiple heavy metal ions by aptamer-based colorimetric assay. Sens. Actuators B Chem..

[38] ams OSRAM (2023). AS7343 14-Channel Multi-Spectral Sensor; Datasheet DS001046, ver. 6-00.

[39] Braga M.S., Jaimes R.F.V.V., Borysow W., Gomes O.F., Salcedo W.J. (2017). Portable multispectral colorimeter for metallic ion detection and classification. Sensors.

[40] Hariharan J., Ampatzidis Y., Abdulridha J. The Basis for Development of a Foundational Biomarker Reflectance Signature Database System for Plant Cell Identification, Disease Detection, and Classification Purposes. Proceedings of the 10th IEEE Annual Computing and Communication Workshop and Conference (CCWC).

[41] Mittal S., Kim J., Bhargava R. (2022). Statistical Considerations and Tools to Improve Histopathologic Protocols with Spectroscopic Imaging. Appl. Spectrosc..

[42] Fei A.T., Strand D.W., Wang J. (2024). Registration of hyperspectral images and mass spectrometry data for the correlation of tissue optical spectra and molecular profiles. Proc. SPIE.

[43] Martínez-Arboleyda D.N., Cruz-Guerrero I.A., Campos-Delgado D.U. (2024). Tumor Tissue Classification in Hyperspectral Histopathology Images Through Individual and Ensemble of Machine Learning Algorithms. XLVI Mexican Conference on Biomedical Engineering.

[44] Durkee M.S., Ai J., Casella G., Cao T., Chang A., Halper-Stromberg A., Jabri B., Clark M.R., Giger M.L. (2024). Pseudo-spectral angle mapping for automated pixel-level analysis of highly multiplexed tissue image data. bioRxiv.

[45] Liberda D., Koziol P., Wróbel T. (2023). Comprehensive Histopathology Imaging in Pancreatic Biopsies: High Definition Infrared Imaging with Machine Learning Approach. Int. J. Biol. Sci..

[46] Tang J., Henderson A., Gardner P. (2021). Exploring AdaBoost and Random Forests machine learning approaches for infrared pathology on unbalanced data sets. Analyst.

[47] Fanjul-Vélez F., Pampín-Suárez S., Arce-Diego J.L. (2020). Application of Classification Algorithms to Diffuse Reflectance Spectroscopy Measurements for Ex Vivo Characterization of Biological Tissues. Entropy.

[48] Nguyen M.H., Zhang Y., Wang F., De La Garza Evia Linan J., Markey M.K., Tunnell J.W. (2021). Machine learning to extract physiological parameters from multispectral diffuse reflectance spectroscopy. J. Biomed. Opt..

[49] Reistad N., Sturesson C. (2022). Distinguishing tumor from healthy tissue in human liver ex vivo using machine learning and multivariate analysis of diffuse reflectance spectra. J. Biophotonics.

[50] Boichenko E., Kirsanov D. (2023). Optical spectroscopy and chemometrics in intraoperative tumor margin assessment. TrAC Trends Anal. Chem..

[51] Sattlecker M., Stone N., Bessant C. (2014). Current trends in machine-learning methods applied to spectroscopic cancer diagnosis. TrAC Trends Anal. Chem..

[52] Witteveen M., Sterenborg M.J.C., Sterenborg H.J.C.M., van Leeuwen T.G. (2022). Comparison of preprocessing techniques to reduce nontissue-related variations in hyperspectral reflectance imaging. J. Biomed. Opt..

[53] Galli M., Zoppis I., De Sio G., Chinello C., Pagni F., Magni F., Mauri G. (2016). A Support Vector Machine Classification of Thyroid Bioptic Specimens Using MALDI-MSI Data. Adv. Bioinform..

[54] Santillan A., Tomas R.C., Bangaoil R., Lopez R.A., Gomez M.H., Ramos E.M., Sumpaico F.D.S., Parungao L.G.O. (2021). Discrimination of malignant from benign thyroid lesions through neural networks using FTIR signals obtained from tissues. Anal. Bioanal. Chem..

[55] Tampu I.E., Eklund A., Johansson K., Gimm O., Haj-Hosseini N. (2023). Diseased thyroid tissue classification in OCT images using deep learning: Towards surgical decision support. J. Biophotonics.

[56] Xanthopoulos P., Pardalos P.M., Trafalis T.B. (2013). Support Vector Machines in Robust Data Mining.

[57] Morais C.L.M., Giamougiannis P., Grabowska R.O., Wood N.J., Martin-Hirsch P.L., Martin F.L. (2020). A three-dimensional discriminant analysis approach for hyperspectral images. Analyst.

[58] Binaghi E., Gallo I., Boschetti M., Brivio P.A. (2004). A neural adaptive model for hyperspectral data classification under minimal training conditions. Proceedings of the Image and Signal Processing for Remote Sensing X.

[59] Li G., Ma S., Li K., Zhou M., Lin L. (2022). Heterogeneity classification based on hyperspectral transmission imaging and multivariate data analysis. Infrared Phys. Technol..

[60] Cebrián P.L., Martín-Pérez A., Villa M., Sancho J., Rosa G., Vázquez G., Sutradhar P., de Ternero A.M., Chavarrias M., Lagares A. (2023). Deep Recurrent Neural Network Performing Spectral Recurrence on Hyperspectral Images for Brain Tissue Classification. Design and Architecture for Signal and Image Processing (DASIP 2023).

[61] Hasbi N.H., Bade A., Chee F.P., Rumaling M.I. (2022). Pattern Recognition for Human Diseases Classification in Spectral Analysis. Computation.

[62] Denkçeken T., Canpolat M., Başsorgun C.İ., Baykara M. Application of principal component analysis and linear discriminant analysis on elastic light single-scattering spectroscopic data for classification of prostate tissue: A preliminary study. Proceedings of the 2014 18th National Biomedical Engineering Meeting (BIYOMUT).

[63] Strickert M., Keilwagen J., Schleif F.-M., Villmann T., Biehl M. (2009). Matrix Metric Adaptation Linear Discriminant Analysis of Biomedical Data. Proceedings of the Bio-Inspired Systems: Computational and Ambient Intelligence (IWANN 2009).

[64] Mansfield J.R., McIntosh L.M., Crowson A.N., Mantsch H.H., Jackson M. (1999). LDA-Guided Search Engine for the Nonsubjective Analysis of Infrared Microscopic Maps. Appl. Spectrosc..

[65] Mu X., Remiszewski S., Kon M.A., Ergin A., Diem M. (2018). Optimizing decision tree structures for spectral histopathology (SHP). Analyst.

[66] Septiana L., Suzuki H., Ishikawa M., Obi T., Kobayashi N., Ohyama N., Wihardjo E., Andiani D. Classification of Elastic and Collagen Fibers in H&E Stained Hyperspectral Images. Proceedings of the 2019 41st Annual International Conference of the IEEE Engineering in Medicine and Biology Society (EMBC).

[67] Martin F., German M.J., Wit E., Fearn T., Ragavan N., Pollock H.M. (2007). Identifying variables responsible for clustering in discriminant analysis of data from infrared microspectroscopy of a biological sample. J. Comput. Biol..

[68] Rinnan Å., van den Berg F., Engelsen S.B. (2009). Review of the most common pre-processing techniques for near-infrared spectra. TrAC Trends Anal. Chem..

[69] Trendafilov N.T., Gallo M. (2021). Linear discriminant analysis (LDA). Multivariate Data Analysis on Matrix Manifolds.

[70] Cevikalp H. Theoretical Analysis of Linear Discriminant Analysis Criteria. Proceedings of the 2006 IEEE 14th Signal Processing and Communications Applications.

[71] Sifaou H., Kammoun A., Alouini M.-S. Improved LDA Classifier Based on Spiked Models. Proceedings of the 2018 IEEE 19th International Workshop on Signal Processing Advances in Wireless Communications (SPAWC).

[72] Nibouche O., Asharindavida F., Wang H., Vincent J., Liu J., van Ruth S., Maguire P., Rahman E. (2024). A new sub-class linear discriminant for miniature spectrometer based food analysis. Chemom. Intell. Lab. Syst..

[73] Féret J.-B., Asner G.P., Jacquemoud S. Regularization of discriminant analysis for the study of biodiversity in humid tropical forests. Proceedings of the 2011 3rd Workshop on Hyperspectral Image and Signal Processing: Evolution in Remote Sensing (WHISPERS).

[74] Bamgbade A., Somorjai R.L., Dolenko B., Pranckeviciene E., Nikulin A.E., Baumgartner R. (2005). Evidence Accumulation to Identify Discriminatory Signatures in Biomedical Spectra. Artificial Intelligence in Medicine (AIME 2005).

[75] Huang J.-W., Chen Y.-H., Phoa F.K.H., Lin Y.-H., Lin S. (2024). An efficient approach for identifying important biomarkers for biomedical diagnosis. Biosystems.

[76] Paplomatas P., Kyriakou Z., Anagnostopoulos K., Vrahatis A.G. (2025). Bayesian subset-based gene selection for biomarker discovery in high-dimensional data. Acad. Mol. Biol. Genom..

[77] Pranckeviciene E., Baumgartner R., Somorjai R. Consensus-based identification of spectral signatures for classification of high-dimensional biomedical spectra. Proceedings of the 17th International Conference on Pattern Recognition.

[78] Rajpoot K., Rajpoot N.M. (2004). SVM Optimization for Hyperspectral Colon Tissue Cell Classification. Medical Image Computing and Computer-Assisted Intervention—MICCAI 2004.

[79] Prigent S., Descombes X., Zugaj D., Zerubia J. Spectral analysis and unsupervised SVM classification for skin hyper-pigmentation classification. Proceedings of the 2010 2nd Workshop on Hyperspectral Image and Signal Processing: Evolution in Remote Sensing (WHISPERS).

[80] Tang H.L. (2004). Organ Origin Identification Based on Fine Feature Analysis Using Support Vector Machines. Int. J. Comput. Internet Manag..

[81] Fernández-Ibáñez V., Fearn T., Montañés E., Quevedo J.R., Soldado A., de la Roza-Delgado B. (2010). Improving the Discriminatory Power of a Near-Infrared Microscopy Spectral Library with a Support Vector Machine Classifier. Appl. Spectrosc..

[82] Garcia-Allende P.B., Anabitarte F., Conde O.M., Mirapeix J., Madruga F.J., López-Higuera J.M. (2008). Support Vector Machines in hyperspectral imaging spectroscopy with application to material identification. Proc. SPIE.

[83] Wu D., Ma C. The Support Vector Machine (SVM) Based Near-Infrared Spectrum Recognition of Leaves Infected by the Leafminers. Proceedings of the First International Conference on Innovative Computing, Information and Control—Volume I (ICICIC’06).

[84] Roy A., Chakraborty S. (2023). Support vector machine in structural reliability analysis: A review. Reliab. Eng. Syst. Saf..

[85] Guido R. (2024). An Overview on the Advancements of Support Vector Machine in the Field of Medicine. Information.

[86] Nuzhny A., Lyubynskaya T.E., Shumsky S. (2007). Detection of the cancerous tissue sections in the breast optical biopsy dataflow using neural networks. Proceedings of the 11th Mediterranean Conference on Medical and Biomedical Engineering and Computing 2007.

[87] Soleimanpoor M., Mokhtaridoost M., Gönen M. (2023). A Kernel-Based Multilayer Perceptron Framework to Identify Pathways Related to Cancer Stages. Machine Learning, Optimization, and Data Science.

[88] Mishra P., Passos D., Marini F., Xu J., Amigo J.M., Gowen A.A., Jansen J.J., Biancolillo A., Roger J.-M., Rutledge D.N. (2022). Deep learning for near-infrared spectral data modelling: Hypes and benefits. TrAC Trends Anal. Chem..

[89] Lee Y.J., Park C., Kim H., Cho S.J., Yeo W.-H. (2024). Artificial intelligence on biomedical signals: Technologies, applications, and future directions. Med-X.

[90] Gorishniy Y., Rubachev I., Khrulkov V., Babenko A. (2021). Revisiting Deep Learning Models for Tabular Data. Proceedings of the NIPS’21: 35th International Conference on Neural Information Processing Systems (NeurIPS 2021).

[91] Cui R., Yu H., Xu T., Xing X., Cao X., Yan K., Chen J. (2022). Deep Learning in Medical Hyperspectral Images: A Review. Sensors.

[92] Tampu I.E., Eklund A., Johansson K., Gimm O., Haj-Hosseini N. Classification of thyroid diseases in OCT images using convolutional neural networks. Proceedings of the Advanced Biomedical and Clinical Diagnostic and Surgical Guidance Systems XX.

[93] Hu X., Niu P., Wang J., Zhang X. (2019). A Dynamic Rectified Linear Activation Units. IEEE Access.

[94] Banerjee C., Mukherjee T., Pasiliao E.L. An Empirical Study on Generalizations of the ReLU Activation Function. Proceedings of the 2019 ACM Southeast Conference.

[95] Kulathunga N., Ranasinghe N.R., Vrinceanu D., Kinsman Z., Huang L., Wang Y. (2021). Effects of Nonlinearity and Network Architecture on the Performance of Supervised Neural Networks. Algorithms.

[96] Vargas-Yun V.M., Guijo-Rubio D., Gutiérrez P.A., Hervás-Martínez C. (2021). ReLU-Based Activations: Analysis and Experimental Study for Deep Learning. Advances in Artificial Intelligence.

[97] Banerjee C., Mukherjee T., Pasiliao E.L. The Multi-phase ReLU Activation Function. Proceedings of the 2020 ACM Southeast Conference.

[98] Passos D., Mishra P. (2022). Perspectives on deep learning for near-infrared spectral data modelling. NIR News.

[99] Rizzo R., Dziadosz M., Kyathanahally S.P., Shamaei A., Kreis R. (2023). Quantification of MR spectra by deep learning in an idealized setting: Investigation of forms of input, network architectures, optimization by ensembles of networks, and training bias. Magn. Reson. Med..

[100] Kingma D.P., Ba J. Adam: A Method for Stochastic Optimization. Proceedings of the 3rd International Conference on Learning Representations (ICLR).

[101] Zhang Y., Shen J. Fingerprint Representation of Metabolite Magnetic Resonance Spectroscopy with Deep Learning. Proceedings of the 2024 ISMRM & ISMRT Annual Meeting & Exhibition (ISMRM).

[102] Khashei M., Nazgouei E., Bakhtiarvand N. (2023). Intelligent Discrete Deep Learning Based Classification Methodology in Chemometrics. J. Chem. Inf. Model..

[103] Ennett C.M., Frize M., Scales N.B. Evaluation of the logarithmic-sensitivity index as a neural network stopping criterion for rare outcomes. Proceedings of the 4th International IEEE EMBS Special Topic Conference on Information Technology Applications in Biomedicine.

[104] Tran M.H., Ma L., Little J.V., Chen A.Y., Fei B. (2022). Thyroid carcinoma detection on whole histologic slides using hyperspectral imaging and deep learning. Proc. SPIE.

[105] Bellantuono L., Tommasi R., Pantaleo E., Verri M., Amoroso N., Crucitti P., Di Gioacchino M., Longo F., Monaco A., Naciu A.M. (2023). An eXplainable Artificial Intelligence analysis of Raman spectra for thyroid cancer diagnosis. Sci. Rep..

[106] Gao L., Wu S., Wongwasuratthakul P., Chen Z., Cai W., Li Q., Lin L.L. (2024). Label-Free Surface-Enhanced Raman Spectroscopy with Machine Learning for the Diagnosis of Thyroid Cancer by Using Fine-Needle Aspiration Liquid Samples. Biosensors.

[107] Tran M.H., Ma L., Mubarak H., Gomez O., Yu J., Bryarly M., Fei B. (2024). Detection and Margin Assessment of Thyroid Carcinoma with Microscopic Hyperspectral Imaging. J. Biomed. Opt..

[108] Edwards K.T., Halicek M., Little J.V., Chen A.Y., Fei B. Multiparametric Radiomics for Predicting the Aggressiveness of Papillary Thyroid Carcinoma Using Hyperspectral Images. Proceedings of the SPIE Medical Imaging.

[109] Girolami I., Marletta S., Pantanowitz B., Volmar C.A.M., Juhlin C.C., Mete O. (2020). Impact of image analysis and artificial intelligence in thyroid pathology, with particular reference to cytological aspects. Cytopathology.

[110] Mittal P., Condina M.R., Klingler-Hoffmann M., Kaur G., Oehler M.K., Sieber O.M., Palmieri M., Kommoss S., Brucker S., McDonnell M.D. (2021). Cancer Tissue Classification Using Supervised Machine Learning Applied to MALDI Mass Spectrometry Imaging. Cancers.

[111] Dolezal J.M., Wolk R., Hieromnimon H.M., Howard F.M., Srisuwananukorn A., Karpeyev D., Ramesh S., Kochanny S., Kwon J.W., Agni M. (2023). Deep learning generates synthetic cancer histology for explainability and education. npj Precis. Oncol..

[112] Wemmert C., Krüger J.M., Forestier G., Sternberger L., Feuerhake F., Gançarski P. Stain unmixing in brightfield multiplexed immunohistochemistry. Proceedings of the 2013 IEEE International Conference on Image Processing (ICIP).

[113] Huang L., Luo R., Liu X., Hao X. (2022). Spectral imaging with deep learning. Light Sci. Appl..

